# The Ratios of monounsaturated to saturated phosphatidylcholines in lung adenocarcinoma microenvironment analyzed by Liquid Chromatography-Mass spectrometry and imaging Mass spectrometry

**DOI:** 10.1038/s41598-019-45506-3

**Published:** 2019-06-20

**Authors:** Yusuke Muranishi, Toshihiko Sato, Shinji Ito, Junko Satoh, Akihiko Yoshizawa, Shigeyuki Tamari, Yuichiro Ueda, Yojiro Yutaka, Toshi Menju, Tatsuo Nakamura, Hiroshi Date

**Affiliations:** 1 Department of Thoracic Surgery, Kyoto city Hospital, Kyoto, Japan; 2 Department of General Thoracic Surgery, Breast and Pediatric Surgery, Fukuoka University School of Medicine, Fukuoka, Japan; 3 Medical Research Support Center, Kyoto University Graduate School of Medicine, Kyoto, Japan; 4 Department of Diagnostic Pathology, Kyoto University Graduate School of Medicine, Kyoto, Japan; 5 Department of Thoracic Surgery, Kyoto University Graduate School of Medicine, Kyoto, Japan; 6 Department of Organ and Tissue Reconstruction, Institute for Frontier Life and Medical Sciences, Kyoto University, Kyoto, Japan

**Keywords:** Non-small-cell lung cancer, Lipidomics, Mass spectrometry

## Abstract

Adenocarcinoma is the most common type of lung cancer, and can be classified into various histologic subtypes. However, little is known about the subtype-dependent variations in lipid metabolism processes. We performed dual lipidomic analyses using liquid chromatography–mass spectrometry (LC-MS) and matrix-assisted laser desorption/ionization imaging mass spectrometry (MALDI-IMS) to identify possible biomarkers to distinguish adenocarcinoma specimens from normal lung specimens, and to determine if there are any differences in lipid metabolism among the histologic subtypes (lepidic, acinar, papillary, micropapillary, solid, and mucinous). LC-MS was used to characterize the lipid profiles of lung adenocarcinoma and normal lung tissue, and MALDI-IMS analysis was performed to confirm the results with information on lipid localization within the lung. LC-MS analysis found significant differences in the relative abundances of phosphatidylcholine (PC)(16:0/16:0) (*P* = 0.0432) and sphingomyelin (SM)(42:2) (*P* < 0.0001) between adenocarcinoma and normal lung specimens. The ratios of PC(16:0/16:1)/PC(16:0/16:0), PC(16:0/18:1)/PC(16:0/16:0), and PC(16:0/18:1)/PC(16:0/18:0) were significantly higher in adenocarcinoma specimens (*P* = 0.02221, *P* = 0.0004, and *P* = 0.0215, respectively). MALDI-IMS analysis confirmed that these ratios were significantly higher in adenocarcinoma regions of the lung. The ratio of PC(16:0–18:1)/PC(16:0–18:0) was significantly lower in solid subtypes than in other subtypes (*P* = 0.0028). The monounsaturated/saturated PC ratios may have applications in adenocarcinoma diagnoses and subtyping.

## Introduction

Lung cancer is a major cause of cancer-related death throughout the world, and is often associated with a poor prognosis. Non-small-cell lung carcinomas account for approximately 85% of all lung cancers, and include adenocarcinomas, squamous cell carcinomas, and large-cell carcinomas^1^. Among these, adenocarcinomas represent the most common histologic variant of lung cancer^2^.

In 2011, the International Association for the Study of Lung Cancer, the American Thoracic Society, and the European Respiratory Society collaboratively developed a classification scheme to standardize the diagnostic criteria for lung adenocarcinomas^3^. In this system, lung adenocarcinomas are histologically classified into atypical adenomatous hyperplasia, adenocarcinomas *in situ*, minimally invasive adenocarcinomas, and invasive adenocarcinomas. Invasive adenocarcinomas are further divided into lepidic predominant, papillary predominant, acinar predominant, micropapillary predominant, solid predominant, invasive mucinous, and colloid/fetal/enteric predominant subtypes (Supplementary Table 1). These classifications have demonstrated good correlations with pathological malignancy and patient prognoses^4^.

The metabolic processes of cancer have been extensively investigated^5^. For example, recent studies have reported on the relationship between tumour progression and lipid metabolism^6,7^. In addition, the expression of monounsaturated lipids has been found to be significantly higher than that of saturated lipids in various forms of cancer, including lung cancer^8–11^. Several types of phospholipids have therefore been proposed as candidate biomarkers for lung cancer^12–16^.

However, no reports in the existing literature have described lipid metabolism in lung adenocarcinoma according to the different histologic subtypes. We hypothesized that the different subtypes would have dissimilar lipid metabolic processes due to their variations in prognoses, pathological malignancy, and pathological findings^4^. The aim of this study was to identify a distinct biomarker that can be used to differentiate adenocarcinoma from normal lung tissue. We also investigated if the different adenocarcinoma subtypes have distinctive phospholipid profiles.

In this study, we conducted a comprehensive investigation of the lipid profiles of lung adenocarcinoma and normal lung specimens using liquid chromatography–MS (LC-MS). These results were revaluated and confirmed using matrix-assisted laser desorption/ionization imaging MS (MALDI-IMS) supplemented with information on the lung localization of each lipid.

## Results

### Patient characteristics

The clinicopathological features of the patients are shown in Table 1. Samples from Patients 1 to 6 were subjected to both LC-MS and MALDI-IMS analyses. The majority of patients presented with Stage I tumours. Solid tumours (observed in 8 cases) were the most common of all subtypes. This was followed by papillary (7 cases), invasive mucinous (3 cases), acinar (3 cases), micropapillary (2 cases), and lepidic (2 cases) subtypes.

**Table 1:** Clinicopathological features of the patients.

Patient No.	Age	Histology	T	N	p-Stage, UICC 7th ed.	p-Stage, UICC 8th ed.	Tumour size (mm)
1^a^	71	MP	t1	n0	IA	IA3	22
2^a^	70	Pap	t1	n0	IA	IA3	23
3^a^	70	Solid	t1	n0	IA	IA3	29
4^a^	42	Acinar	t1	n0	IA	IA3	27
5^a^	76	Mucinous	t2	n0	IB	IIA	45
6^a^	76	Pap	t2	n0	IB	IB	39
7	66	Acinar	t2	n0	IB	IB	34
8	46	Acinar	t2	n2	IIIA	IIIA	38
9	73	Lep	t1	n0	IA	IA2	18
10	66	Lep	t1	n0	IA	IA3	22
11	72	MP	t1	n0	IA	IA3	24
12	82	Mucinous	t1	n0	IA	IA2	12
13	65	Mucinous	t4	n0	IIIB	IIIA	80
14^b^	64	Pap	t1	n0	IA	IA3	26
15	64	Pap	t1	n0	IA	IA3	25
16	54	Pap	t1	n0	IA	IA2	17
17	72	Pap	t1	n0	IA	IA2	20
18	60	Pap	t1	n1	IIA	IA3	27
19	70	Solid	t1	n0	IA	IA2	15
20	62	Solid	t1	n0	IA	IA2	20
21	75	Solid	t1	n0	IA	IA3	21
22	77	Solid	t2	n0	IB	IIA	43
23	58	Solid	t2	n0	IB	IIA	40
24	74	Solid	t1	n1	IIA	IIB	23
25	72	Solid	t2	n1	IIB	IIB	38

^a^Denotes patients who underwent both liquid chromatography–mass spectrometry and imaging mass spectrometry analyses.

^b^Samples from Patient 14 were used in the histological analysis, electron microscope imaging, and liquid chromatography–mass spectrometry and imaging mass spectrometry analyses shown in Figs 3 and 4.

MP = Micropapillary. Pap = Papillary. Lep = Lepidic. UICC = Union for International Cancer Control.

### LC-MS analysis results

**Figure 1:**
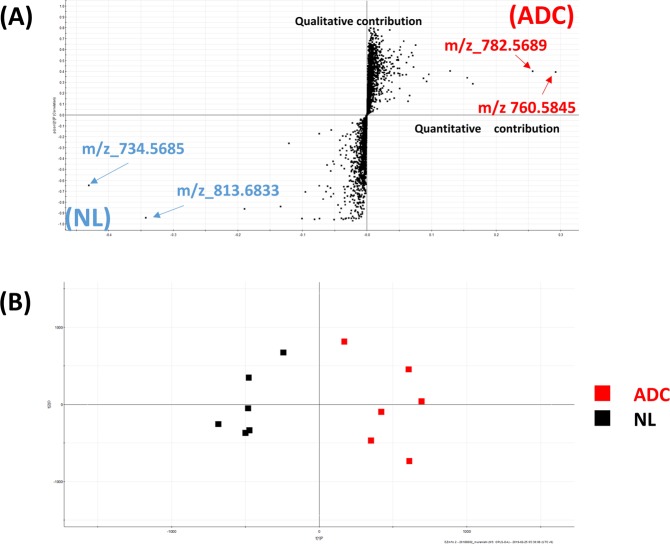
(**A**) S-plot of the orthogonal projection to latent structures discriminant analysis of adenocarcinoma (ADC) and normal lung (NL) specimens. The X-axis and Y-axis show the degree of quantitative contributions and qualitative contributions, respectively. Positive values are more representative of ADC specimens and negative values are more representative of NL specimens. (**B**) Score-plot of the orthogonal projection to latent structures discriminant analysis of adenocarcinoma (ADC) and normal lung (NL) specimens.

The S-plot of the orthogonal projections to latent structures discriminant analysis (OPLS-DA) revealed abundant ions at *m/z* 782.57 and *m/z* 760.58 in the adenocarcinoma specimens, whereas ions at *m/z* 734.57 and *m/z* 813.68 were more abundant in the normal lung specimens (Fig. 1A) We show the scores plot for OPLS-DA (Fig. 1B). Progenesis QI software predicted that these ions are derived from lipid species that are generally abundant in lung specimens^12,14,17,18^. We show the top four variable importance in projection (VIP) scores of the OPLS-DA and the corresponding m/z (Table 2). The scores were calculated by EZinfo software (Waters). The highest VIP score was 23.9762 (for the ion with m/z of 734.568 and retention time 8.23 min), followed by 19.7024 (m/z of 813.68 and retention time 13.51 min), 15.1571 (m/z of 760.58 and retention time 8.51 min) and 14.1025 (m/z of 782.56 and retention time 6.66 min). We further examined these lipid species through authentic MS/MS spectral analyses using the same LC-MS system. As summarized in Supplementary Table 2 and Supplementary Fig. 1A–F, the Progenesis QI predictions were confirmed by the authentic MS/MS spectral analyses. By matching the retention times and MS/MS fragmentation patterns with those of standard compounds, the ions at *m/z* 734.57 (Supplementary Fig. 1A) and *m/z* 760.58 (Supplementary Fig. 1B) were identified to be the protonated ions of PC (16:0/16:0) and PC (16:0/18:1), respectively. The retention time of ions at *m/z* 782.57 was close to that of other PCs, and the MS/MS fragmentation pattern matched that of PC (36:4) (Supplementary Fig. 1C). The retention time of ions at *m/z* 813.68 was close to the elution range for sphingomyelin (SM) under the same LC conditions, and its MS/MS fragmentation pattern matched that of SM (42:2) (Supplementary Fig. 1D). We also examined other ions (*m/z* 762.60 and *m/z* 732.55) that were abundant in the lung sample. By matching the retention times and MS/MS fragmentation patterns with those of standard compounds (Supplementary Fig. 1E), the ions at *m/z* 762.60 were identified to be PC (16:0/18:0). The retention time of ions at m/z 732.55 was close to that of other PCs, and the MS/MS fragmentation pattern matched that of PC (16:0/16:1) (Supplementary Fig. 1F).

**Table 2:** Variable importance in projection (VIP) score of the OPLS-DA in the top four ions

m/z	Retention time (min)	VIP score
734.57	8.24	23.9762
813.68	13.51	19.7024
760.58	8.51	15.1571
782.57	6.66	14.1025

VIP, variable importance in projection.

We also picked up several insignificant lipids identified by the global metabolomic analysis with LC–MS and summarised them in the Supplemental Table 3. The VIP scores of these candidate lipids are low, and there is almost no difference in the average signal intensity between normal lung and lung adenocarcinoma tissues. However, we did not use standard compounds to identify these insignificant lipids.

**Figure 2:**
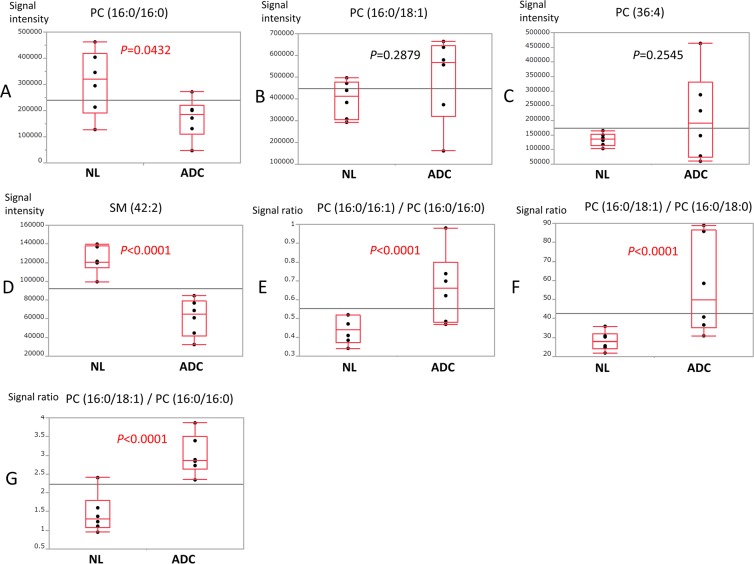
Box plots showing the relative abundance of each lipid (**A**–**D**) and the ratios of monounsaturated phosphatidylcholine (PC) to saturated PC (**E**–**G**) in adenocarcinoma (ADC) and normal lung (NL) specimens from 6 patients through liquid chromatography–mass spectrometry analysis. *P* values were calculated using Student’s *t*-test.

There were significant differences between the adenocarcinoma and normal lung specimens in the relative abundances of PC (16:0/16:0) and SM (42:2), especially in the latter (*P* < 0.0001). The mean abundances of both lipids were higher in the normal lung specimens than the adenocarcinoma specimens. In contrast, there were no significant differences between the 2 groups in the mean abundances of PC (16:0/18:1) (*P* = 0.2879) and PC (36:4) (*P* = 0.2454) (Fig. 2A–D). As shown in Fig. 2E,F, the ratios of PC (16:0/16:1)/PC (16:0/16:0), PC (16:0/18:1)/PC (16:0/16:0), and PC (16:0/18:1)/PC (16:0/18:0) were significantly higher in the adenocarcinoma specimens than the normal lung specimens (*P* = 0.02221, *P* = 0.0004, and *P* = 0.0215, respectively).

### MALDI-IMS analysis results

**Figure 3:**
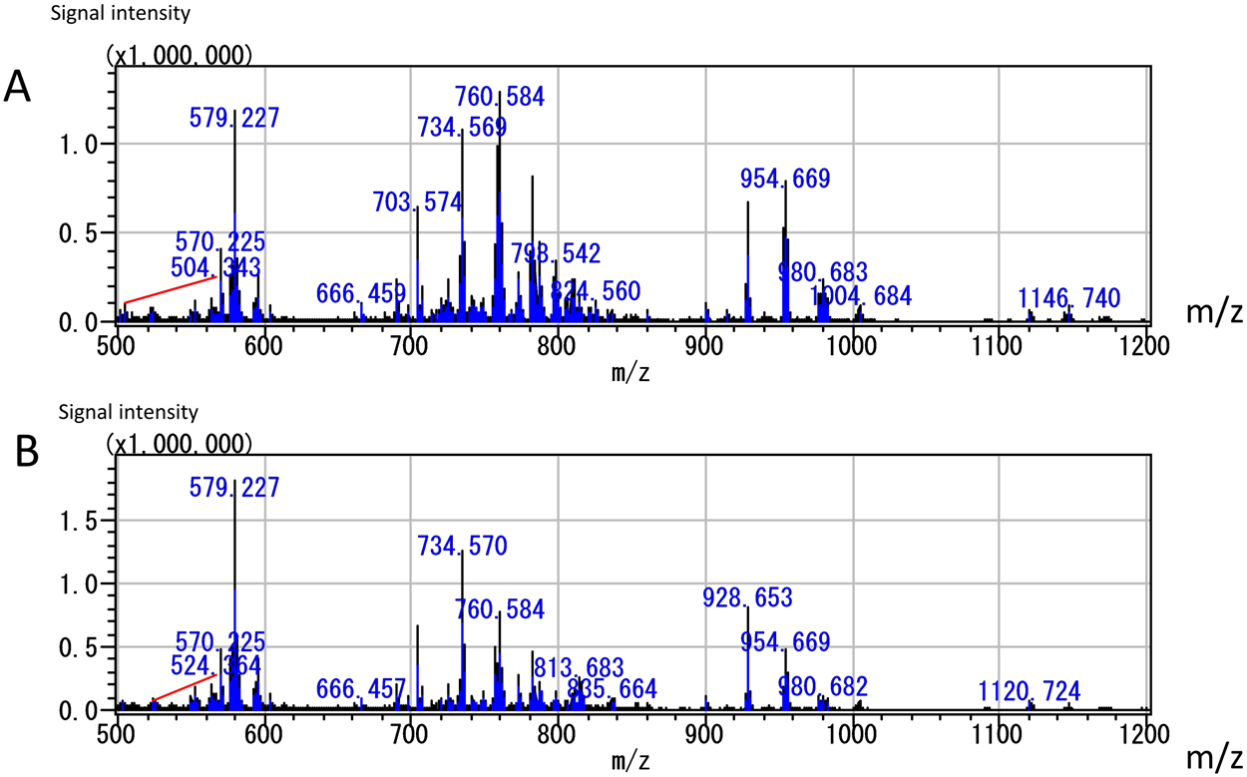
Typical mass spectra of samples from adenocarcinoma (**A**) and normal lung specimens (**B**). The data shown in this figure were obtained from Patient 14 in Table 1.

**Figure 4:**
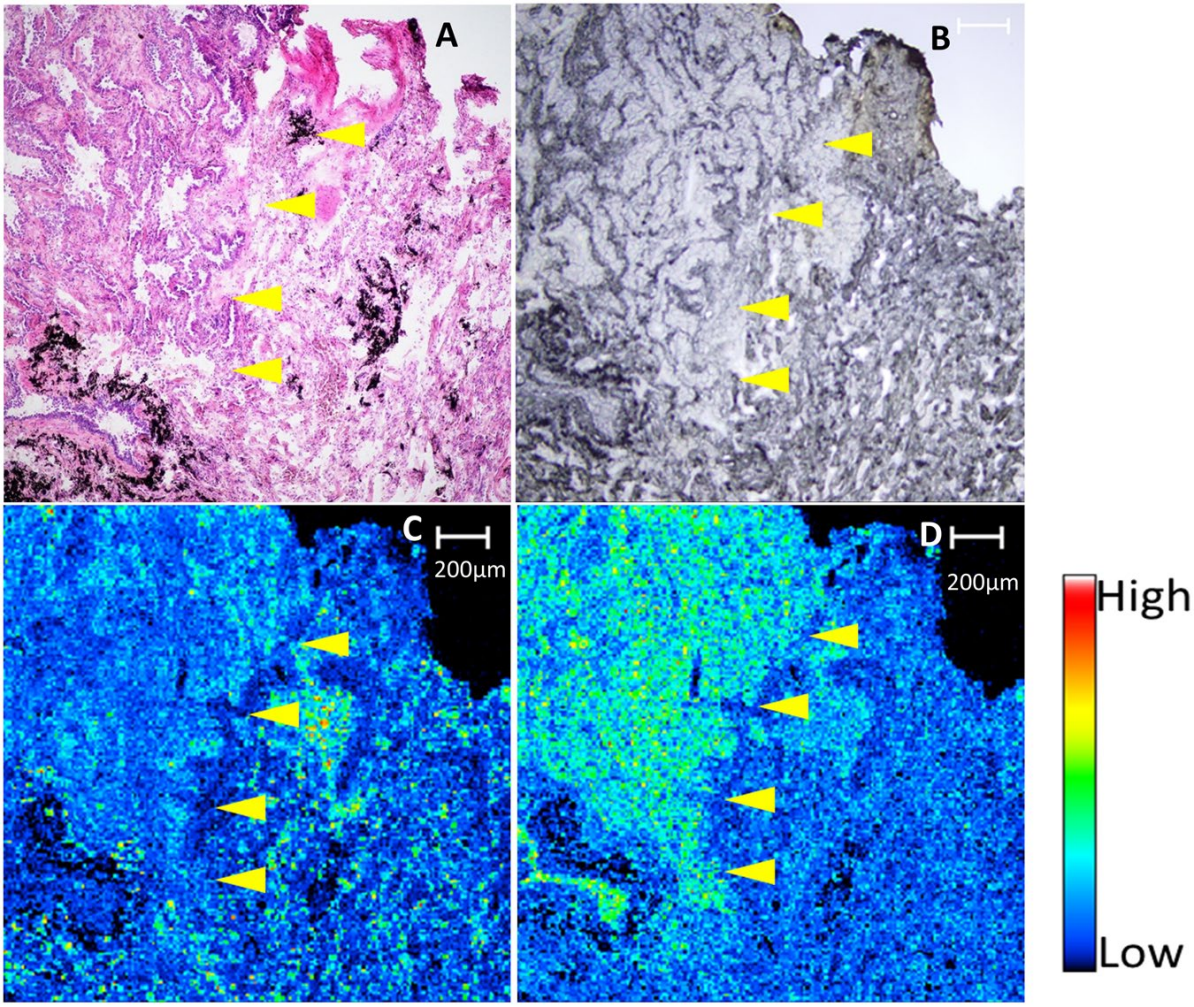
Histological image with haematoxylin and eosin staining (**A**), electron microscope image (**B**), and matrix-assisted laser desorption/ionization imaging mass spectrometry images (**C**,**D**) of adjacent or near-adjacent sections from a single patient (Patient 14 in Table 1). (**C**) shows an ion image of phosphatidylcholine (PC) (16:0/16:0), and Fig. 4d shows an ion image of PC (16:0/18:1). Haematoxylin and eosin staining indicated papillary adenocarcinoma in the left side of the histological image (**A**), and the right side was identified as normal lung tissue. PC (16:0/16:0), which was the most abundant lipid in the normal lung region, was concentrated along the alveolar wall (**C**). In contrast, PC (16:0/18:1), which was the most abundant lipid in the tumour region, was concentrated among the tumour cells (**D**). Bar: 200 μm. The boundary between the tumour tissue and the normal tissue is pointed by yellow arrow head.

The intra-lung distribution of the various lipid species and the ratios of their abundances were examined using MALDI-IMS. The typical mass spectra of samples from adenocarcinoma and normal lung specimens are presented in Fig. 3. The samples shown in Figs 3 and 4 were collected from Patient 14 (shown in Table 1).

Figure 4A shows the different distribution of ions between the tumour region and surrounding normal lung parenchyma as revealed by haematoxylin and eosin (H&E) staining. Figure 4B shows an electron microscope image of the same area. We present MALDI-IMS images from adjacent or near-adjacent sections of ions at *m/z* 734.57 (PC [16:0/16:0]) in Fig. 4C and at *m/z* 760.58 (PC [16:0/18:1]) in Fig. 4D. Supplementary Fig. 2 shows MALDI-IMS images of ions at *m/z* 732.55 (PC [16:0/16:1]), *m/z* 762.60 (PC [16:0/18:0]), *m/z* 782.57 (PC [36:4]), and *m/z* 813.68 (SM [42:2]).

**Figure 5:**
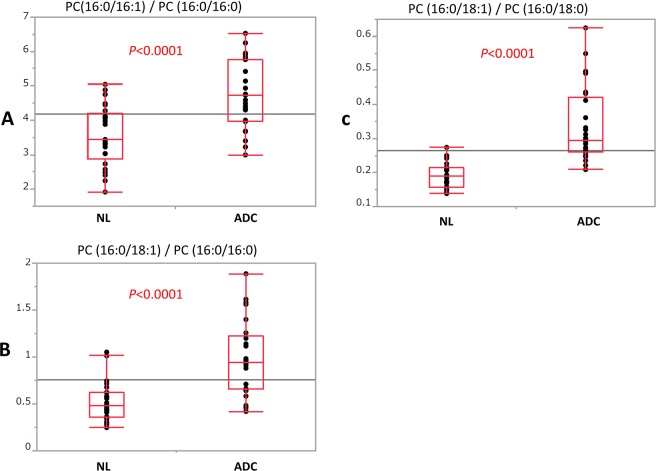
Box plots of the ratios of monounsaturated phosphatidylcholine (PC) to saturated PC (**A**–**C**) between adenocarcinoma (ADC) and normal lung (NL) specimens. *P* values were calculated using Student’s *t*-test.

As shown in Fig. 5, the intensity ratios of PC (16:0/16:1)/PC (16:0/16:0), PC (16:0/18:1)/PC (16:0/16:0), and PC (16:0/18:1)/PC (16:0/18:0) were significantly higher (*P* < 0.0001) in the adenocarcinoma specimens than the normal lung specimens, which was consistent with the results from the LC-MS analysis.

**Figure 6:**
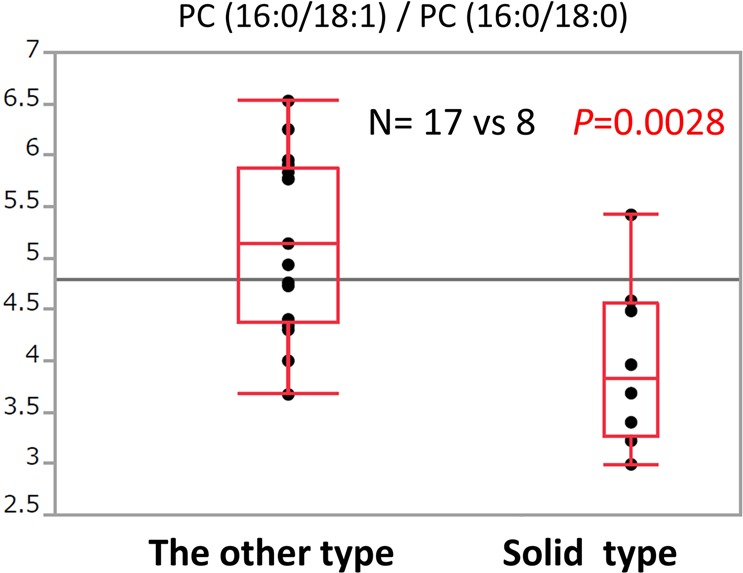
Box plot of the ratio of phosphatidylcholine (PC) (16:0/18:1) to PC (16:0/18:0) between solid adenocarcinoma and other subtypes. The *P* value was calculated using Student’s *t*-test.

The intensity ratio of PC (16:0/18:1)/PC (16:0/18:0) was significantly lower in solid predominant tumour specimens than the other subtypes (n = 8 vs 17; *P* = 0.0028) (Fig. 6).

## Discussion

Cancer-associated metabolic processes have been the subject of numerous studies since it was discovered that cancer cells demonstrate an upregulation of glycolysis and lactate production^19^. Many types of cancer show increased glucose uptake and enhanced glycolytic rates, which suggests that the rapid growth of tumour cells contributes to metabolic alterations. In lung cancer, the activation of lipid metabolism has been suggested to be a hallmark of carcinogenesis^10,12,14,20^.

First, we performed global metabolome analysis (LC–MS) using three cases and three controls (the total sample size was 6) and identified the molecules with m/z of 734.57, 813.68, 760.58 and 782.56 to be significant candidates. The molecule with m/z of 760.58 was identified to be a protonated ion of PC (16:0/18:1). Because previous studies have reported increases in PC (16:0/18:1) in lung cancer cells^10,12,14^, we closely observed PC (16:0/18:1). From the standard deviation and mean difference of m/z760.58 using three cases and three controls (the total sample size was 6), we confirmed that the total sample size we have used (N = 12, six cases and six controls) was acceptable at least for the comparison of the candidate molecules.

In this study, we conducted an in-depth comparative investigation of the lipid profiles of lung adenocarcinoma and normal lung specimens using LC-MS to shed light on the metabolic processes in the lung cancer microenvironment. In the S-plot shown in Fig. 1, PC (16:0/16:0) and SM (42:2) were found to be abundant in normal lung specimens, whereas PC (16:0/18:1) and PC (36:4) were more abundant in lung adenocarcinoma specimens.

We also found that PC (16:0/16:0) and SM (42:2) were significantly less abundant in all lung adenocarcinoma specimens than in normal lung tissue (Fig. 2A,D).

Pulmonary surfactant, a complex of lipids and proteins secreted by type II alveolar cells, reduces the surface tension at the air-liquid interface^17,18,21^. This surfactant consists of PC, SM, and various surfactant proteins (e.g., SP-A, SP-B, SP-C, and SP-D). PC (16:0/16:0), or dipalmitoylphosphatidylcholine, is a main component of pulmonary surfactant^17,18^. We posit that a reduction in the number of normal type II alveolar cells in the tumour region may have contributed to the observed reduction of PC (16:0/16:0) in the adenocarcinoma specimens.

Our experiments detected a prominent difference in the abundance of SM (42:2) between adenocarcinoma and normal lung specimens (*P* < 0.0001). This suggests the presence of a specialized metabolic pathway for SM (42:2) in lung cancer cells. SM, which is the major sphingolipid in mammalian cells and represents one of the main lipid components of pulmonary surfactant, has also been reported to decrease in other tumour cells^22^. SM also has shown an anti-cancer effect in colon cancer^23^. Based on our findings, we believe that decreases in the abundance of PC (16:0/16:0) and SM (42:2) may have useful applications in the auxiliary diagnosis of adenocarcinoma. These 2 lipids may therefore play important roles as key lipids in cancer metabolism. On the other hand, we found no significant differences in the mean abundance of PC (16:0/18:1) (*P* = 0.2879) and PC (36:4) (*P* = 0.2454) between adenocarcinoma and normal lung specimens (Fig. 2B,C). In contrast, previous studies have reported increases in PC (16:0/18:1) in lung cancer cells^10,12,14^. We were unable to identify the reasons for this discrepancy, and further analyses are needed to determine if these 2 lipids have potential applications in cancer diagnoses.

The levels of monounsaturated fatty acids and monounsaturated PCs have been reported to be significantly higher than those of saturated and polyunsaturated PCs in the cancer microenvironment^8–11^. Therefore, our study focused on the ratios of PC (16:0/16:1)/PC (16:0/16:0), PC (16:0/18:1)/PC (16:0/16:0), and PC (16:0/18:1)/PC (16:0/18:0). Our experiments showed that these ratios were significantly higher in the lung adenocarcinoma specimens than the normal lung specimens. The detection of increases in these ratios may therefore have useful applications in the auxiliary diagnosis of cancer.

MALDI-IMS is an MS technique that facilitates the acquisition of mass spectra directly from specimens and constructs density maps of the detected ions. While conventional MS analyses require lipid extraction, MALDI-IMS does not involve the destruction of histological structures in specimens. In addition, analysts are able to compare the MALDI-IMS images with corresponding optical images^24,25^. MALDI-IMS is therefore commonly used to investigate the localization of lipids in cancer specimens. In this study, we considered MALDI-IMS to be the best tool to examine lipid distribution in the adenocarcinoma specimens, which demonstrate various pathological characteristics. Similar to the results of the LC-MS analysis, the MALDI-IMS analysis found significantly higher signal intensity ratios of PC (16:0/18:1)/PC (16:0/18:0), PC (16:0/16:1)/PC (16:0/16:0), and PC (16:0/18:1)/PC (16:0/16:0) in the lung adenocarcinoma specimens than the normal lung specimens. The results from the 2 analytical approaches indicated that lung adenocarcinoma specimens have a higher expression of monounsaturated PCs than normal lung specimens. In this way, the LC-MS results were revaluated and confirmed using the MALDI-IMS analysis, which was supplemented with information on intra-lung localization of each lipid.

Although it is said our MALDI-IMS instrument has the ability to perform MS/MS analysis, we could not get MS/MS information of MALDI-IMS in this study. Interfering noises were mixed in the background during MS/MS analysis, and it was extremely difficult to identify clear MS/MS fragmentation patterns. We could not clarify the reason why MS/MS analysis using MALDI-IMS did not work. It might be controversial whether the results of the LC–MS/MS and MALDI-IMS analyses are in perfect correspondence. However, we believe that their correspondence is highly likely because of the following: (1) We used the same specimens. (2) The observed accurate masses were identical for corresponding molecules. (3) The characteristics and abundance of the corresponding molecules are very similar. They all were quite abundant in lung specimens and very easily and strongly ionised.

**Figure 7:**
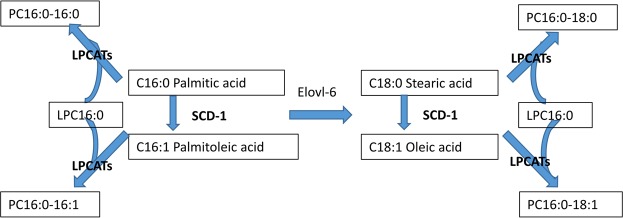
Presumed schematic diagram of phosphatidylcholine (PC) development. Monounsaturated fatty acids are endogenously produced from the corresponding saturated fatty acids through the action of stearoyl-CoA desaturase-1 (SCD-1). Lyso-PC acyltransferase (LPCAT) may catalyse the synthesis of PC in pulmonary surfactant and play a critical role in respiratory physiology. Elongation of very long fatty acids 6 (ELOVL6) is a rate-limiting enzyme that catalyses the elongation reaction of C16:0 to C18:0.

Analysis of the adenocarcinoma subtypes revealed that the PC (16:0/18:1)/PC (16:0/18:0) ratio was significantly lower in solid type tumours than in the other subtypes (*P* = 0.0028). We suspect that this difference is influenced by enzymes that play an important role in PC synthesis. PCs are synthesized via *de novo* and remodelling pathways^11,26^. Figure 7 shows a schematic diagram of PC development. In the remodelling pathway, monounsaturated fatty acids (palmitoleic acid [16:1] and oleic acid [18:1]) are endogenously produced from their corresponding saturated fatty acids (palmitic acid [16:0] and stearic acid [18:0]) through the action of stearoyl-CoA desaturase-1 (SCD-1), and subsequently converted into monounsaturated PC. SCD-1 is thought to play an important role in cancer progression^27^. In addition, lyso-PC acyltransferases (LPCATs) and elongation of very long fatty acids (ELOVLs) have also been reported to be important components of PC metabolism^11,16,26,28^. LPCATs, which catalyse the reacylation of phospholipids, are particularly important in PC synthesis. ELOVL6 is a rate-limiting enzyme that catalyses the elongation reaction of palmitic acid [16:0] to stearic acid [18:0]^16^. Although it is difficult to determine the reason for the different PC (16:0/18:1)/PC (16:0/18:0) ratio between solid tumours and the other subtypes, we posit that the difference may be driven by the aforementioned enzymes.

### Limitations

First, there was a relatively small number of samples in this study. Specifically, the sample size for each adenocarcinoma subtype was small, and there was a bias in the number of samples in each subtype group. We also have the advanced cases in our sample, although most samples were from pathological Stage I. There may be also some individual differences of lipids metabolism among different patients. We could not clearly identify the reliability using multivariate analysis, and we did not practicality validate the cross-validation or data training model in this small number of groups. Therefore, future studies should be conducted using larger sample sizes for each subtype and pathological Stage.

Second, several different key enzymes are involved in the metabolic pathways of phospholipids. As this study focused on comparing the abundance of metabolic products between normal lung and lung adenocarcinoma specimens, we remain uncertain as to which enzymes are actually active in these metabolic pathways in adenocarcinoma tissue. This mechanism should be further examined using additional experiments (such as immune-histochemical validation) to elucidate the roles of enzymes such as SCD-I, LPCATs, and ELOVL6.

Finally, it is quite difficult to precisely discriminate the positions of double bonds in acyl groups by MS/MS fragmentation using the LC–MS system because of the similar fragmentation patterns and close retention times. The names of the lipids in our identification results lack the position information of the double bonds. The positions of acyl-sn would play an important role in cancer phenotype, and it would be the subject of a future study.

## Conclusion

The lung adenocarcinoma microenvironment is characterized by significantly higher ratios of monounsaturated PCs to saturated PCs, and the solid tumour subtype possibly had a lower ratio than the other adenocarcinoma subtypes. Despite the above limitations, we believe that our study will be important in the field of cancer metabolism.

## Materials and Methods

### Clinical specimens

Samples of lung adenocarcinoma and normal lung tissue were obtained from patients who underwent lung cancer surgery in the Department of Thoracic Surgery, Kyoto University Hospital (Kyoto, Japan) between January 2013 and December 2014. All samples were collected in accordance with ethical guidelines, and all patients were approached based on approved ethical guidelines. Written informed consent was obtained from all patients prior to inclusion in the study (Authorization Number: R0097). A total of 25 patients who underwent pulmonary resection for lung adenocarcinoma with curative intent were included in analysis. Twenty-five patients were included to ensure that each invasive adenocarcinoma subtype (lepidic, acinar, papillary, micropapillary, solid, and mucinous) was represented for the pathological diagnosis.

Adenocarcinoma and normal lung specimens (each several square millimetres in size) were harvested from these patients and flash frozen in liquid nitrogen. The specimens were stored at −80 °C until used in the experiments. Normal lung specimens were harvested from parts of the lung distant from the tumour within the same lobe. All H&E-stained slides were evaluated by a pathologist and a thoracic surgeon to determine the subtype of adenocarcinoma. These slides were used as a guide for MALDI-IMS analysis.

### LC-MS analysis

The frozen specimens were excised using a disposable scalpel and weighed after freezing. Total lipids were extracted using a commercial lipid extraction kit (BioVision Inc, Milpitas, CA, USA). The volume of extraction solution was determined according to the measured weight of each specimen. The volume-matched lipid extracts for each specimen were aliquoted and dried under nitrogen before being reconstituted in a mixture of 50% isopropanol/25% acetonitrile/25% water. The reconstituted samples were separated using an ultra-high performance liquid chromatography H-class system (Waters, Milford, MA, USA) with a binary gradient pump. The gradient program was as follows: 40% to 43% B in 2 min (Curve 6), 43% to 50% B in 0.1 min (Curve 1), 50% to 54% B in 9.9 min (Curve 6), 54% to 70% B in 0.1 min (Curve 1), 70% to 99% B in 5.9 min (Curve 6), 99% to 40% B in 0.1 min (Curve 6), and 40% B for 1.9 min. Mixtures of 0.1% formic acid/10 mM ammonium formate/60% acetonitrile and 0.1% formic acid/10 mM ammonium formate/10% acetonitrile/90% isopropanol were used as mobile phases A and B, respectively. The flow rate was set to 0.4 mL/min. The eluates from ultra-high performance liquid chromatography were infused online to a Xevo G2-S Q-Tof mass spectrometer equipped with an ESI ion source (Waters). MS was performed in the electrospray ionization positive ion mode. For global lipid profiling, datasets were obtained using MS^E^ Technology (Waters), in which the MS and MS/MS spectra were acquired globally in a single run. Collision-induced dissociation was utilised for the authentic MS/MS spectral analyses. Datasets were acquired with precursor m/z values and fixed collision energies optimal for each compound. The optimal collision energies for each compound were determined through the stepwise screening of the voltages (from 10 to 200 V). The datasets obtained from the global lipid profiling (Six cases; pairs of 6 adenocarcinoma specimens and 6 normal lung specimens) were exported to Progenesis QI software (Nonlinear Dynamics, New Castle upon Tyne, UK) for analysis. Alignments, peak findings, relative abundance calculations, and database searches of LIPID MAPS [https://www.lipidmaps.org/] and the Human Metabolome Database [http://www.hmdb.ca/] were performed using Progenesis QI. The peak areas for each compound were normalized to total compound intensity and examined as relative abundances. The normalized relative abundances of all compounds were exported to EZinfo (Umetrics, Umea, Sweden). OPLS-DA was performed using EZinfo, and the obtained result was converted to the S-plot format. The reliability of compound identification by Progenesis QI was revaluated using the authentic MS/MS spectral analyses in which standard compounds (if commercially available) were measured by the same LC-MS system with appropriate modifications to the MS settings. Lipid standards (PC [16:0/16:0], PC [16:0/18:1], and PC [16:0/18:0]) were purchased from Avanti Polar Lipids (Alabaster, AL, USA).

### MALDI-IMS analysis

The frozen specimens were sectioned on a cryostat (CM1850; Leica, Wetzler, Germany) at −20 °C. Cryosections of 8-μm thickness were mounted onto indium–tin oxide slides, and every other section was mounted onto common glass slides for H&E staining. The slides for adenocarcinoma specimens and normal lung specimens were prepared separately. This process was entrusted to the Kyoto Nutrition Pathology Laboratory. Each section was coated with 9-aminoacridine hemihydrate (Acros Organics, Geel, Belgium), which served as the matrix for MALDI-IMS. The slides were anchored in a vacuum deposition chamber (SVC-700TM/700-2; Sanyu Electron, Tokyo, Japan) and mechanically coated with 9-aminoacridine hemihydrate evaporated at −20 °C. The time required for this vapor deposition process was 8 min.

High-resolution MALDI-IMS analysis was performed on a high-resolution microscopic imaging mass spectrometer (RK27-4050C; Shimadzu, Kyoto, Japan; the prototype model of IMScope) equipped with a 355-nm Nd:YAG laser. MS data were acquired in positive ion mode in the 500.0–1200.0 *m/z* range using an external calibration method. Each region of interest was determined from the microscopic view of the slides and referring to the adjacent H&E-stained sections. We selected regions of interest that were mainly occupied by one subtype (>50%); mass spectra were obtained at a spatial resolution of 10 μm.

The spectra and mean signal intensities of the regions of interest were analysed, and the MS data were normalized to the total ion current to eliminate variations in ionization efficiency using the Imaging MS Solution™ program (Shimadzu).

### Statistical analysis

The difference in signal intensities or signal intensity ratios were compared using Student’s *t*-test. Statistical analyses were performed using JMP version 12.0 (SAS, Cary, NC, USA), and *P* values below 0.05 were considered statistically significant.

### Ethical statement

All study protocols were approved by the Ethics Committee for Clinical Research, Kyoto University Hospital (Approval number: R369).

## Supplementary information

Supplementary information

## References

[1] Ferlay J (2015). Cancer incidence and mortality worldwide: sources, methods and major patterns in GLOBOCAN 2012. Int J Cancer.

[2] Herbst Roy S., Heymach John V., Lippman Scott M. (2008). Lung Cancer. New England Journal of Medicine.

[3] Travis WD (2011). International association for the study of lung cancer/american thoracic society/european respiratory society international multidisciplinary classification of lung adenocarcinoma. J Thorac Oncol.

[4] Yoshizawa A (2011). Impact of proposed IASLC/ATS/ERS classification of lung adenocarcinoma: prognostic subgroups and implications for further revision of staging based on analysis of 514 stage I cases. Mod Pathol.

[5] Schulze A, Harris AL (2012). How cancer metabolism is tuned for proliferation and vulnerable to disruption. Nature.

[6] Menendez JA, Lupu R (2007). Fatty acid synthase and the lipogenic phenotype in cancer pathogenesis. Nature Reviews Cancer.

[7] Zaidi N (2013). Lipogenesis and lipolysis: the pathways exploited by the cancer cells to acquire fatty acids. Prog Lipid Res.

[8] Ide Y (2013). Human breast cancer tissues contain abundant phosphatidylcholine(36ratio1) with high stearoyl-CoA desaturase-1 expression. PLoS One.

[9] Roongta UV (2011). Cancer cell dependence on unsaturated fatty acids implicates stearoyl-CoA desaturase as a target for cancer therapy. Mol Cancer Res.

[10] Guo S, Wang Y, Zhou D, Li Z (2014). Significantly increased monounsaturated lipids relative to polyunsaturated lipids in six types of cancer microenvironment are observed by mass spectrometry imaging. Sci Rep.

[11] Kurabe N (2013). Accumulated phosphatidylcholine (16:0/16:1) in human colorectal cancer; possible involvement of LPCAT4. Cancer Sci.

[12] Marien E (2015). Non-small cell lung cancer is characterized by dramatic changes in phospholipid profiles. Int J Cancer.

[13] He M, Guo S, Ren J, Li Z (2016). *In Situ* Characterizing Membrane Lipid Phenotype of Human Lung Cancer Cell Lines Using Mass Spectrometry Profiling. J Cancer.

[14] Lee GK (2012). Lipid MALDI profile classifies non-small cell lung cancers according to the histologic type. Lung Cancer.

[15] Merino Salvador M (2017). Lipid metabolism and lung cancer. Crit Rev Oncol Hematol.

[16] Eyra Marien1, M. M. *et al*. Phospholipid profiling identifies acyl chain elongation as a ubiquitous trait and potential target for the treatment of lung squamous cell carcinoma. *Oncotarget* Vol. 7 (2016). 10.18632/oncotarget.7179 PMC4914306 26862848

[17] Chen X, Hyatt BA, Mucenski ML, Mason RJ, Shannon JM (2006). Identification and characterization of a lysophosphatidylcholine acyltransferase in alveolar type II cells. Proc Natl Acad Sci USA.

[18] Kurabe N (2013). Visualization of phosphatidylcholine (16:0/16:0) in type II alveolar epithelial cells in the human lung using imaging mass spectrometry. Pathol Int.

[19] Warburg O (1956). On the origin of cancer cells. Science.

[20] Xie W, Gao D, Jin F, Jiang Y, Liu H (2015). Study of Phospholipids in Single Cells Using an Integrated Microfluidic Device Combined with Matrix-Assisted Laser Desorption/Ionization Mass Spectrometry. Anal Chem.

[21] Nakanishi H (2006). Cloning and characterization of mouse lung-type acyl-CoA:lysophosphatidylcholine acyltransferase 1 (LPCAT1). Expression in alveolar type II cells and possible involvement in surfactant production. J Biol Chem.

[22] Barcelo-Coblijn G (2011). Sphingomyelin and sphingomyelin synthase (SMS) in the malignant transformation of glioma cells and in 2-hydroxyoleic acid therapy. Proc Natl Acad Sci USA.

[23] Lemonnier LA (2003). Sphingomyelin in the suppression of colon tumors: prevention versus intervention. Archives of Biochemistry and Biophysics.

[24] Kawashima M (2013). High-resolution imaging mass spectrometry reveals detailed spatial distribution of phosphatidylinositols in human breast cancer. Cancer Sci.

[25] Goto T (2014). The expression profile of phosphatidylinositol in high spatial resolution imaging mass spectrometry as a potential biomarker for prostate cancer. PLoS One.

[26] Harayama T (2014). Lysophospholipid acyltransferases mediate phosphatidylcholine diversification to achieve the physical properties required *in vivo*. Cell Metab.

[27] Huang, J *et al*. SCD1 is associated with tumor promotion, late stage and poor survival in lung adenocarcinoma. *Oncotarget*, **Vol**. **7**, **No**. **26** (May 19, 2016). 10.18632/oncotarget.9461 PMC5129985 27223066

[28] Shindou H, Shimizu T (2009). Acyl-CoA:lysophospholipid acyltransferases. J Biol Chem.

