# Supplementary material for: The Ratios of monounsaturated to saturated phosphatidylcholines in lung adenocarcinoma microenvironment analyzed by Liquid Chromatography-Mass spectrometry and imaging Mass spectrometry
Source: Sci Rep. 2019 Jun 20;9:8916. doi: 10.1038/s41598-019-45506-3 (PMC6586780; doi:10.1038/s41598-019-45506-3)
Supplement: Supplementary file 1 — Supplementary information [file 41598_2019_45506_MOESM1_ESM.pdf]

Supplementary Information for: The Ratios of monounsaturated to saturated phosphatidylcholines in lung adenocarcinoma microenvironment analyzed by Liquid Chromatography-Mass spectrometry and imaging Mass spectrometry

Yusuke Muranishi [1 2], Toshihiko Sato[1 5*], Shinji Ito[3], Junko Satoh[3], Akihiko Yoshizawa[4], Shigeyuki Tamari[1 2], Yuichiro Ueda[1 2], Yojiro Yutaka[1 2], Toshi Menju[1], Tatsuo Nakamura[1 2], Hiroshi Date[1]

Authors' affiliations

[1] Department of Thoracic Surgery, Kyoto University Graduate School of Medicine, Kyoto, Japan

[2] Department of Organ and Tissue Reconstruction, Institute for Frontier Life and Medical Sciences, Kyoto University, Kyoto, Japan

[3] Medical Research Support Center, Kyoto University Graduate School of Medicine, Kyoto, Japan

[4] Department of Diagnostic Pathology, Kyoto University Graduate School of Medicine, Kyoto, Japan

[5]Department of General Thoracic Surgery, Breast and Pediatric Surgery, Fukuoka University School of Medicine, Fukuoka, Japan

***Corresponding author:** Toshihiko Sato

Department of Thoracic Surgery, Kyoto University Graduate School of Medicine, 54 Shogoin-Kawahara-cho, Sakyo-ku, Kyoto 606-8507, Japan

E-mail: tsato@kuhp.kyoto-u.ac.jp

Phone: +81-75-751-4975; Fax: +81-75-751-4974

Supplementary Table 1. International multidisciplinary classification (2011) of lung adenocarcinoma developed by the International Association for the Study of Lung Cancer/American Thoracic Society/European Respiratory Society.

**Preinvasive lesions**
Atypical adenomatous hyperplasia
Adenocarcinoma in situ (≤3 cm)
Non-mucinous and/or mucinous
**Minimally invasive adenocarcinoma**
≤3-cm lepidic predominant tumour with ≤5mm invasion
Non-mucinous and/or mucinous
**Invasive adenocarcinoma**
Lepidic predominant
Acinar predominant
Papillary predominant
Micropapillary predominant
Solid predominant with mucin
**Variants**
Invasive mucinous adenocarcinoma and mixed mucinous/non-mucinous
Colloid
Foetal
Enteric

Supplementary Table 2. Results of MS/MS spectral analyses.

m/z	Lipids	Analytical standard	Retention time (min)
732.55	PC (16:0/16:1)	No	6.60 and 6.76
734.57	PC (16:0/16:0)	Yes	8.33
760.58	PC (16:0/18:1)	Yes	8.59
762.60	PC (16:0/18:0)	Yes	11.04
782.57	PC (36:4)	No	6.76
813.68	SM (42:2)	No	13.56

Collision Energy was 100 V in all cases.

Supplementary Table 3. Insignificant lipids identified by LC–MS analysis

m/z	Average abundance of tumour	Average abundance of normal lung tissue	VIP score	Candidate lipids
732.62	1084.1	1118.5	0.169184	PC (34:1)
772.58	106.7	120.4	0.0810629	PC (35:2)<br>PE (38:2)
752.52	21.1	28.7	0.149077	PC (34:5)<br>PE (37:5)
716.52	120.6	123.6	0.0161938	PE (34:2)<br>PC (31:2)
754.57	69.4	61.4	0.0537465	PE (38:4)<br>PC (35:4)
748.62	164.2	156.1	0.0775411	PC (34:0)<br>PE (37:0)
752.52	138.8	110.9	0.134519	PC (34:5)<br>PE (37:5)
766.53	26.1	25.7	0.00348706	PC (35:5)<br>PE (38:5)

VIP score= variable importance in projection score

PC= phosphatidylcholine PE= phosphatidylethanolamine

*Standard compounds were not used to confirm the identification of these insignificant lipids.

Supplementary Figure 1A

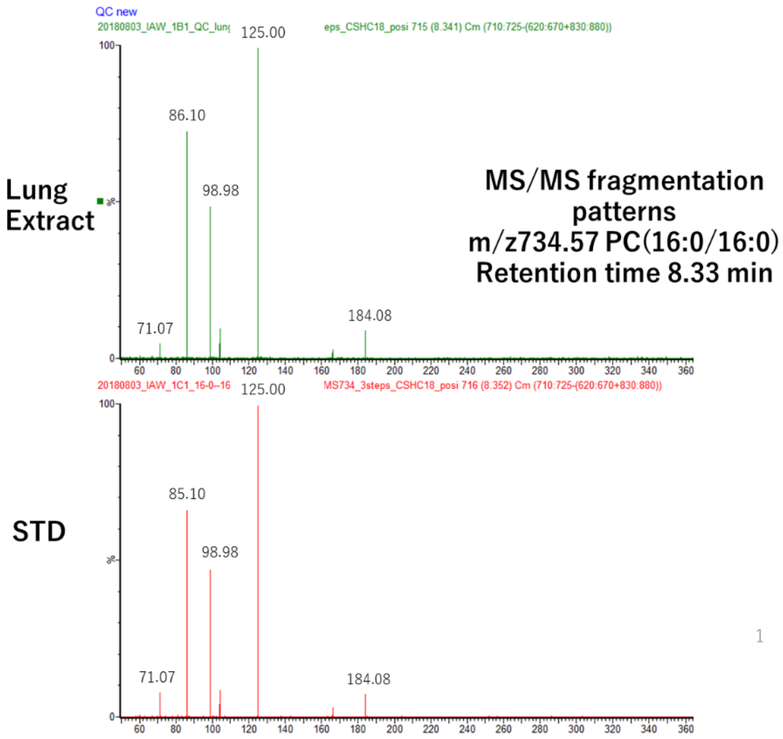

Supplementary Figure 1B

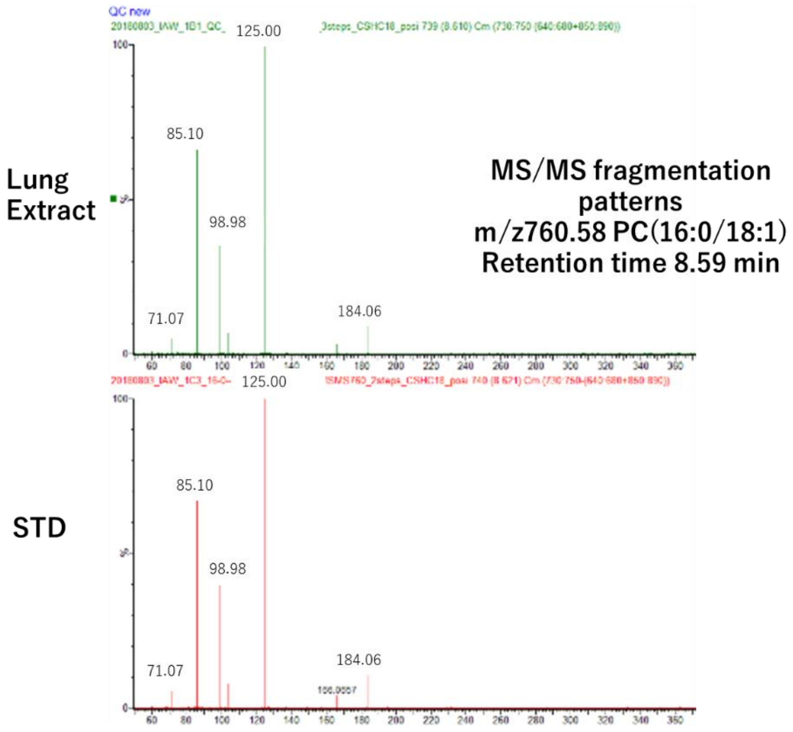

Supplementary Figure 1C

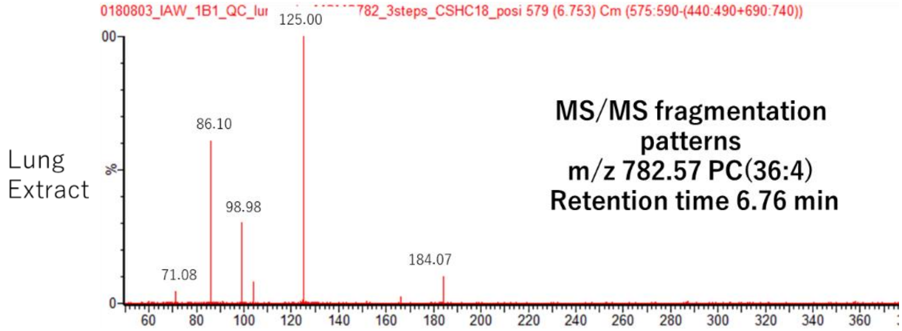

Supplementary Figure 1D

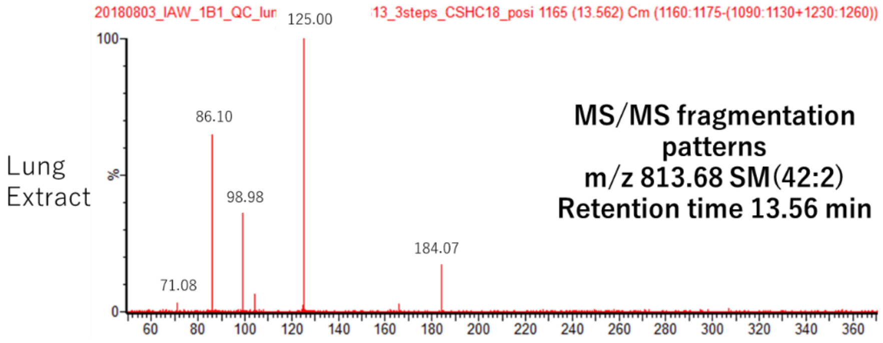

Supplementary Figure 1E

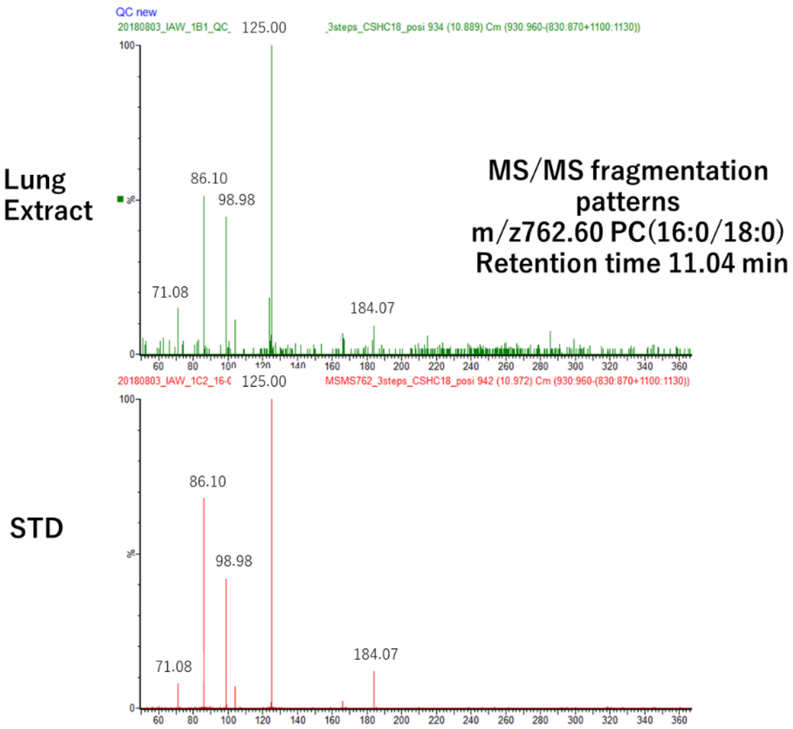

Supplementary Figure 1F

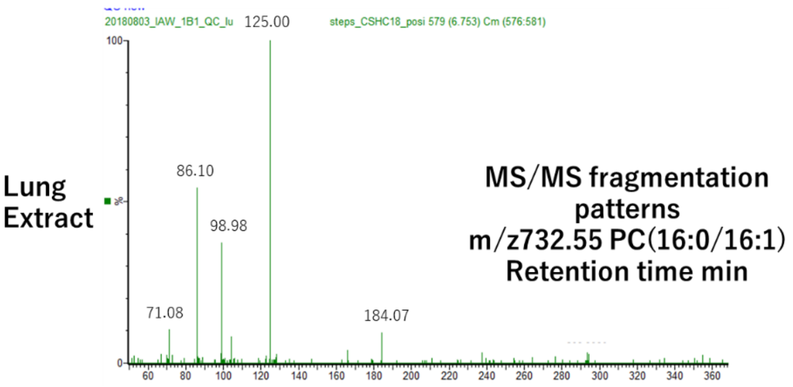

Supplementary Figure 2

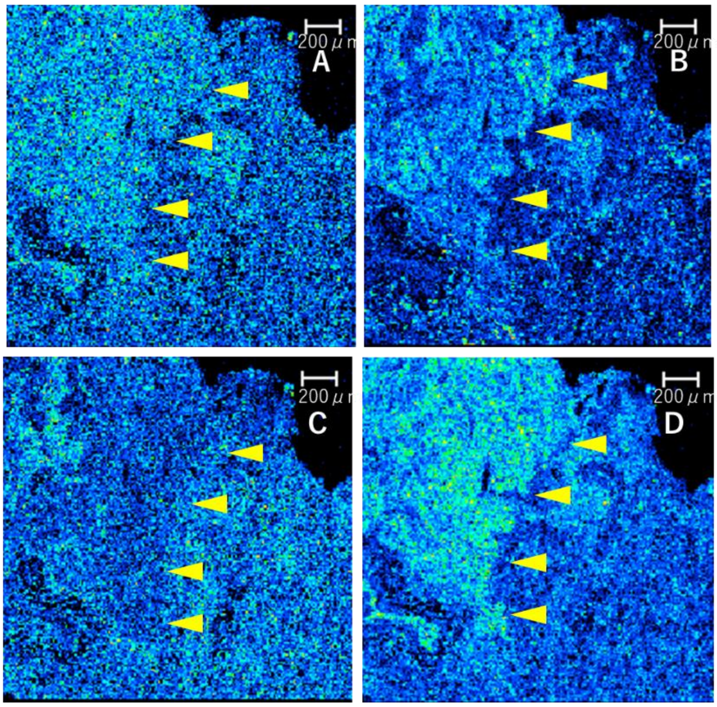

MALDI-IMS images of m/z 762.60 (PC [16:0/18:0]) (A), m/z 732.55 (PC [16:0/16:1]) (B), m/z 813.68 (SM [42:2]) (C), and m/z 782.57 (PC [36:4]) (D). Bar: 200μm.

The boundary between the tumour tissue and the normal tissue is pointed by yellow arrow head.
